# Ostracism Experiences of Sexual Minorities: Investigating Targets’ Experiences and Perceptions by Others

**DOI:** 10.1177/01461672241240675

**Published:** 2024-04-03

**Authors:** Christiane M. Büttner, Selma C. Rudert, Sven Kachel

**Affiliations:** 1 University of Basel, Switzerland; 2 University of Kaiserslautern-Landau, Germany; 3 University of Helsinki, Finland

**Keywords:** LGB, sexual orientation, ostracism, social exclusion

## Abstract

Lesbian, gay, and bisexual (LGB) people face frequent discrimination, maltreatment, and violence for transgressing gender roles upheld in heteronormative societies. Ostracism (i.e., being excluded and ignored) is likely another, understudied form of discrimination against sexual minorities. In a multi-method approach using a nationally representative panel (*N* = 4104) and experience sampling data (*N* = 467, 14 days, *k* = 926 ostracism experiences), we find that LGB individuals report more ostracism experiences than straight individuals. In line with the idea that ostracism toward sexual minorities occurs as a function of gender role nonconformity, lesbians and gay men are rated by an independent rater sample as more likely to be ostracized (*k* = 10,760 ratings) when they are also rated as more lesbian/gay and less gender role conforming. Our findings speak in favor of ostracism as a discriminatory experience of LGB individuals that is driven by transgressions of heteronormativity.

People who identify as part of a sexual minority (e.g., lesbian, gay, and bisexual) face a range of discriminatory behaviors such as rejection, harassment, and even violent assault (e.g., DeSouza et al., 2017; Fasoli et al., 2017; Gordon & Meyer, 2007; Hebl et al., 2002; Katz-Wise & Hyde, 2012). As a result, sexual minorities experience more mental illness (e.g., Meyer, 2003) and attempt suicide twice as often as their straight counterparts (e.g., King et al., 2008). Previous research has been largely silent about the role of ostracism, being excluded and ignored (Williams, 2009), in the everyday life of sexual minorities. Ostracism may be a specific, yet more subtle, form of discrimination against sexual minorities (e.g., DeSouza et al., 2017). Even though targets of ostracism are not physically harmed, ostracism causes the affected individuals a tremendous amount of psychological pain that is neurologically similar to experiencing physical pain (e.g., Eisenberger, 2012; Eisenberger et al., 2003). As a consequence, ostracism bears immense individual and societal costs such as lowering well-being, reducing productivity, and increasing burnout, mental illness, and suicidality (e.g., Chen et al., 2020; Qian et al., 2019; Rudert et al., 2021).

While sexual orientation has been largely ignored as a potential driving factor of ostracism, exclusion from society characterizes the history of sexual minorities: Lesbians, gay men, and bisexual people^1^ (from here-on: LGB) have been stigmatized as having a disease and excluded from society as a consequence: For instance, they were denied the same rights as heterosexual individuals regarding marriage, adoption, or donating blood (e.g., Hammack & Cohler, 2011; Hurley, 2009; Peng & Salter, 2021). LGB people are also ostracized by law in different countries, such as being imprisoned, excommunicated from church, or banned from serving in the military (International Lesbian, Gay, Bisexual, Trans, and Intersex Association, 2016; Pirlott & Cook, 2018). Exclusion is also reflected in invisibility, marginalization, and underrepresentation in the public sphere, constituting psychologically impactful forms of ostracism (e.g., Büttner & Rudert, 2022; Jauch et al., 2023; Lutz et al., 2023; McCarty et al., 2022): Concretely, LGB individuals face underrepresentation in media portrayals (e.g., McInroy & Craig, 2017), in political decision-making processes as parliamentarians (e.g., Magni & Reynolds, 2021), and in educational contexts (e.g., Ellis & High, 2004). These experiences likely contribute to LGB individuals’ overall higher feelings of ostracism compared with heterosexual individuals. Indeed, a recent study documents that Chinese LGB individuals’ feelings of being excluded by society predict higher emotional distress, however, LGB individuals’ feelings were not compared with those of heterosexual individuals (Cheung & Tsang, 2023).

Fortunately, blatant discrimination against LGB individuals is increasingly unacceptable (e.g., Preuß et al., 2020; Steffens, 2005). However, with increasing restriction of blatant discrimination, subtle forms of discrimination—such as ostracism—may increase (e.g., Preuß et al., 2020; Robinson et al., 2013). While many forms of discrimination are characterized by negative attention being directed at the target, ostracism is characterized by withdrawing attention from a target, which makes it both more subtle to detect and to call out. In line with this, in a field study of job applications, lesbian/gay applicants were not blatantly discriminated (e.g., insulted) but fewer words were exchanged with them and interactions with them were generally shorter than for heterosexual applicants (Hebl et al., 2002)—a form of ostracism. Importantly, because of its subtlety, ostracism may constitute a discriminatory behavior that allows the perpetrator to avoid appearing guilty. After all, the reasons for ostracism are often unclear to the target and sources can easily attribute ostracism to socially acceptable reasons (Robinson et al., 2013). For instance, the interviewers in the Hebl et al. (2002) study could have claimed that not bias but mere chance led to shorter exchanges with lesbian/gay applicants. While shorter exchanges likely hindered lesbian/gay applicants’ hiring chances, the interviewers can hardly be held accountable for exchanging fewer words with them. Moreover, societal ostracism such as underrepresentation in policy-making (e.g., Magni & Reynolds, 2021) is even less obviously attributable to a single culprit, and therefore harder to combat than outright discrimination or violence. Engaging in subtle forms of discrimination, such as ostracizing sexual minority members, may also prevent individuals from having to recognize their own biases and instead protect a favorable self-image (“Of course I would never discriminate against the gay colleague, I just forgot to invite him for lunch!,” cf. literature on microaggressions, Nadal et al., 2016).

Importantly, not receiving attention —ostracism— may be just as hurtful if not more than receiving negative attention: Being rendered meaningless and not worthy of attention is a unique component of experiencing ostracism compared with other forms of psychological maltreatment that makes ostracism especially aversive (e.g., Rudert et al., 2017; Williams & Nida, 2009). Experimental studies suggest that being ignored even has stronger psychological consequences than being outrightly rejected (Rudert et al., 2017) and ostracism also elicits stronger negative emotions than bullying (Zadro et al., 2005). Targets of long-term ostracism even report that they would choose physical abuse over being ostracized because they would at least be acknowledged by others then (Williams & Zadro, 2005). In addition, targets of long-term ostracism report that bystanders do not empathize with them and they hope others would empathize more with targets of physical abuse than with those of ostracism (Williams & Zadro, 2005). Crucially, empathy gaps in observers of repeated adversity predict lower helping intentions and may thus further harm targets of ostracism (Zagefka, 2022). In conclusion, ostracism is more subtle, less easy to detect, and thus less easy to combat than other forms of discrimination. In addition, the consequences of ostracism may surmount those of direct rejection or bullying.

Despite the apparent relevance of ostracism for LGB individuals, research on ostracism experiences of LGB individuals is scarce and seems to be limited to occupational contexts. For instance, LGB employees are more likely to be excluded from work events than heterosexual employees (Hoel et al., 2017) and one in five LGB physicians reports feeling ostracized by co-workers (Eliason et al., 2011). Moreover, consequences of ostracism for LGB individuals may potentially be deadly: Among young LGB individuals, losing friends after disclosing one’s sexual orientation, an extreme form of ostracism, is one of the strongest predictors of suicide attempts (Hershberger et al., 1997).

But what may be the mechanisms underlying the relation between one’s sexual orientation and ostracism? We argue that transgressions of heteronormativity are likely to predict ostracism experiences of LGB individuals. First, LGB individuals’ sexual orientation may be perceived as different from that of a heteronormative society and LGB individuals may be excluded as a result, especially when they are perceived as deviating more strongly from the heteronormative standard. For instance, meta-analytic findings suggest that those who deviate from the majority are rejected as a function of the size of the minority (Tata et al., 1996). LGB individuals’ experiences of ostracism may thus depend on perceptions of being different from their social environment. Furthermore, in line with heteronormative gender belief systems (Kite & Deaux, 1987), LGB individuals may be perceived to violate gender role norms (i.e., by not following traditional gender roles, for example, Gordon & Meyer, 2007; Kachel et al., 2016; Morgenroth et al., 2024) and norm violations are a strong predictor of individuals’ decisions to ostracize others (Rudert et al., 2023). Gender role nonconforming behaviors may include, among others, the ways that LGB individuals talk, move, or dress (e.g., Gordon & Meyer, 2007; Kachel et al., 2018; Munson & Babel, 2007). In heteronormative societies, people may react with feelings of threat to individuals who challenge gender roles and the gender binary (e.g., Morgenroth & Ryan, 2021; Van Der Toorn et al., 2020). Feelings of threat in turn increase negative attitudes toward sexual minorities (e.g., Glick et al., 2007). The degree of gender role nonconformity has also been linked to experiences of queerphobic discrimination and violence (e.g., Gordon & Meyer, 2007; Salter & Liberman, 2016). These findings suggest that LGB individuals who conform to gender roles to a lesser extent may be perceived as deviating norm violators which may lead to ostracism. To examine whether norm violations indeed play a role in driving LGB individuals’ ostracism experiences, we examine perceptions of one’s own sexual orientation as different and gender role (non)conformity.

In sum, the present contribution sets out to answer the following questions:

**Research Question 1 (RQ1):** Do LGB individuals experience ostracism more frequently than heterosexual individuals?
**Research Question 2 (RQ2):** Are transgressions of heteronormativity (i.e., perceiving one’s sexual orientation as different and gender role nonconformity) associated with more frequent ostracism of LGB people?

## The Present Research

To paint a full picture of LGB individuals’ ostracism experiences, we use a multi-method approach: Taking a target perspective (i.e., those who get ostracized), Studies 1 and 2 examine self-reported ostracism experiences of LGB (versus straight) individuals in a nationally representative multiwave panel from Germany (Study 1) and highly externally valid experience sampling data in another context, the United States (Study 2). Study 2 also tests whether seeing one’s own sexual orientation as different is a better predictor of ostracism frequency than mere self-categorization of sexual orientation. Complementing the approach of Studies 1 and 2, Study 3 investigates whether others perceive LGB individuals to face ostracism more frequently—a proxy for ostracizability (as a target attribute) which is connected to targets’ ostracism experiences (e.g., Rudert et al., 2023; Rudert, Keller, et al., 2020). Analyzing target experiences *and* perceptions by others is indispensable for understanding the role of norm violations (see, for example, Rudert et al., 2023; Rudert, Keller, et al., 2020) since only assessing one perspective may misrepresent the role of norm violations in ostracism (see, for example, Study 1 vs. 2 in Rudert et al., 2023). Moreover, targets self-report could be prone to biases, such as perceptual defense (i.e., avoiding the recognition of potentially threatening stimuli; Howie, 1952). For cross-validation, Study 3 uses naturally occurring subtle cues (e.g., full-body pictures, faces, and voices) derived from women and men varying in sexual orientation to investigate perceived ostracism (i.e., perceived likelihood that others will ostracize a target person) based on targets’ sexual orientation. Assessing perceived ostracism among raters in a way that does not reflect one’s *own* decision, but *others’* behavior toward the target is important: In general, ostracizing others has psychological costs, and most people condemn ostracizing others (e.g., Rudert et al., 2018). Indicating which targets might get ostracized *by others*, instead of indicating whom one *wants* to ostracize, circumvents that individuals may not want to admit ostracism intentions in a more direct way (e.g., Rudert, Keller, et al., 2020). Finally, Study 3 further reduces social desirability by not mentioning targets’ sexual orientation, incorporates perceived sexual orientation and gender role nonconformity as judgment dimensions in addition to perceived ostracism, avoids spillover effects by using different samples rating each judgment dimension, considers psychosocial variables of raters, and employs a highly controlled experimental approach.

All studies’ procedures were approved by the University of Basel’s (#002-16-1 and #002-16-6) and the University of Kaiserslautern-Landau’s (#364) Institutional Review Boards. Data for Study 1 is subject to German data protection laws, but can be made available to all researchers by the German Institute for Economic Research (https://www.diw.de/en/diw_02.c.222829.en/access.html). The analysis code and materials for Study 1, as well as materials, analysis code, and data for Studies 2 and 3 are available via OSF (https://osf.io/kxsym/). Study designs, hypotheses, sample size, inclusion/exclusion criteria, and analysis plans of the experimental studies were pre-registered on AsPredicted (Study 2 study procedure: https://aspredicted.org/THK_7DZ, Study 2 specific hypotheses: https://aspredicted.org/RGM_PLS, Study 3: https://aspredicted.org/MMM_XYG). We report all manipulations, measures, and exclusions relevant for the presented studies.^2^

As effect size measures, we report Cohen’s *d* for *t*-tests, correlation coefficients *r* for correlations, and regression coefficients (*b*s) for regression analyses, throughout the manuscript. To compare the effects of sexual orientation on ostracism frequency, we additionally report η^2^ for these analyses in Studies 1 and 2.

## Study 1

Study 1 used panel data from the innovation sample of the socio-economic panel (SOEP-IS, Goebel et al., 2019), a nationally representative survey of German adults. We tested whether individuals who identify as LGB report more ostracism experiences than heterosexual individuals (H1). Hypotheses will be numbered consecutively throughout the manuscript.

### Method

#### Participants

The SOEP-IS waves 2015, 2018, and 2022 include measures of ostracism and sexual orientation that 2,609 unique participants answered (see Table 1).

**Table 1.** Descriptive Statistics for Study 1 and Study 2.

	Study 1 (survey year)			Study 2 (study phase)	
Sample charcteristic	2015	2018	2022	Pre-screening	Experience sampling
Sample size	2,264	1,021	819	467	304
Sexual orientation					
Heterosexual	2,214 (97.79%)	1,005 (98.43%)	802 (97.79%)	357 (76.44%)	231 (75.99%)
Lesbian/gay	19 (0.84%)	7 (0.69%)	3 (0.37%)	46 (9.85%)	33 (10.86%)
Bisexual	31 (1.37%)	9 (0.88%)	14 (1.71%)	64 (13.70%)	40 (13.16%)
Gender					
Woman	1,183 (52.27%)	542 (53.09%)	407 (49.69%)	226 (48.39%)	145 (47.70%)
Man	1,081 (47.73%)	479 (46.91%)	394 (48.11%)	229 (49.04%)	151 (49.67%)
Nonbinary	-	-	-	12 (2.57%)	8 (2.63%)
Chose not to answer	-	-	18 (2.20%)	0	0
Age: *M, SD*, Range	50.65, 18.11, 17-96	54.47, 18.42, 17-97	56.22, 17.77, 18-90	36.94, 12.92, 18-75	38.36, 13.08, 18-75
Ostracism frequency: *M, SD*, Range					
Full sample	1.75, 0.76, 1-7	1.60, 0.70, 1-5.75	1.73, 0.70, 1-6	2.57, 1.50, 1-7	3.06, 3.81, 0-26
Heterosexual	1.74, 0.76, 1-7	1.60, 0.70, 1-5.75	1.72, 0.70, 1-6	2.50, 1.48, 1-7	2.82, 3.39, 0-17
Lesbian/gay	1.72, 0.73, 1-3	1.89, 0.91, 1-3	1.50, 0.70, 1.25-1.75	2.80, 1.57, 1-6.25	3.82, 5.41, 0-26
Bisexual	2.04, 0.72, 1-3.5	2.06, 0.79, 1-3.5	1.99, 0.75, 1-3.75	2.81, 1.56, 1-7	3.82, 4.43, 0-17

*Note*. The SOEP-IS (Study 1) assessed gender as a binary variable. Ostracism experiences were assessed before sexual orientation in both studies. We report all sexual orientation items, including all answer options, on the OSF Supplement 1.

#### Measures

Ostracism frequency was measured with the Ostracism Short Scale (Rudert, Keller, et al., 2020): “In the last two months, how often have you experienced the following?” with respect to: “Other people have ignored me,” “Other people have shut me out from conversations,” “Other people have treated me as if I was not there,” “Other people have excluded me from activities,” 1 = *never*, 7 = *always*. Sexual orientation was measured with one item (see OSF Supplement 1). Based on the small subgroup sizes (see Table 1), sexual orientation was dichotomized (LGB vs. heterosexual).

### Results

**Figure 1.**
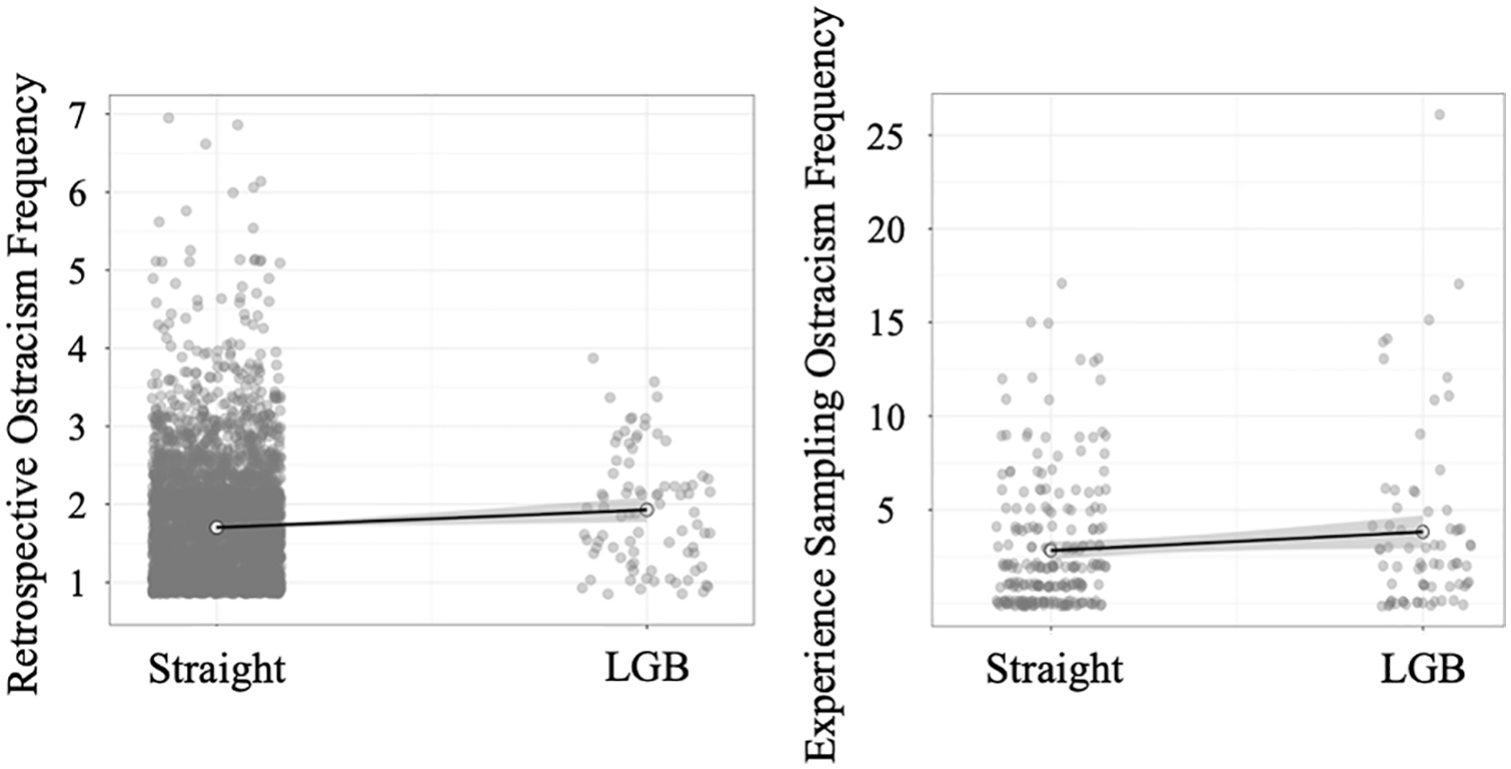
Sexual Orientation Predicting Retrospective Ostracism Frequency in Study 1 (Left Graph) and Experience Sampling Ostracism Frequency in Study 2 (Right Graph). *Note.* Gray areas around regression lines represent standard errors. Lighter points are means.

Descriptively, across all waves, LGB individuals reported higher ostracism frequency (*M* = 1.93, *SD* = 0.74) than heterosexual individuals (*M* = 1.70, *SD* = 0.73). To account for the nested data structure, we computed a mixed model with random intercepts for participant and survey year and found a significant effect of sexual orientation on ostracism, *b* = .18, 95% CI [.02, .34], *p* = .030, η^2^ = .001, see Figure 1 (left graph). In addition, we used Ho et al.’s (2011) *MatchIt* R-package for propensity score matching to estimate the effect of sexual orientation on ostracism frequency. We chose this approach to make the different groups more comparable to test the impact of single attributes (here: sexual orientation) without confusing them with other group differences (here: gender, age, and household income). Propensity scores were estimated using one-to-one nearest-neighbor matching. Because of missing values on income, we were able to match *N* = 64 LGB individuals to heterosexual individuals, achieving a good balance between the two groups, all standardized mean differences >.10. We computed a weighted linear regression with cluster-robust variance to estimate the standard error. Compared with their matched straight counterparts, ostracism frequency was 0.36 scale points higher among LGB individuals, 95% CI [.12, .61], *p* = .004, η^2^ = .07.

### Discussion

LGB individuals reported more frequent ostracism experiences than heterosexual individuals, even when they were matched on demographic dimensions important to the experience of ostracism (e.g., age; Albath et al., 2023). While a difference of 0.23 (matched: 0.36) on a 7-point scale may seem small, previous literature has associated even small increases in ostracism frequency with the development of suicidal ideation and clinically diagnosed depression (Chen et al., 2020; Rudert et al., 2021). However, ostracism experiences that are assessed retrospectively are potentially affected by recall biases. In addition, very few individuals in the SOEP self-identified as LGB. The SOEP assures participants’ anonymity; however, data are collected in a household-based interview style (Goebel et al., 2019) which may contribute to underreporting of queer identities. Moreover, data were confined to one context: Germany. We address these concerns in Study 2, using a smartphone-based, completely anonymous, experience sampling approach and an U.S. American sample. Moreover, being LGB may be an entirely different experience based on one’s social environment. For instance, being the only LGB person in one’s peer group versus feeling like an accepted member of the queer community may affect individuals’ ostracism experiences to a great extent. We therefore address seeing one’s own sexual orientation as different from that of others as a possible predictor of ostracism experiences in the next study.

## Study 2

Study 2 again tests the hypothesis that individuals who identify as LGB report more ostracism experiences than heterosexual individuals (H1), assessed retrospectively, as well as in experience sampling reports of ostracism. We also pre-registered the following hypothesis: Participants who self-report their sexual orientation to deviate more (vs. less) from others will report more ostracism (H2). Data collection was conducted in two phases: A pre-screening where eligibility for participation and trait measures were assessed, and a 14-day experience sampling phase during which participants reported whenever they felt ostracized in daily life.

### Method

In total, 467 U.S. residents completed the pre-screening and reported ostracism using the same Ostracism Short Scale (Rudert, Keller, et al., 2020) as in Study 1. Participants indicated their sexual orientation with one item and, in line with Study 1, sexual orientation was dichotomized (see OSF Supplement 1 for response options and Table 1 for descriptive statistics). Participants also indicated their agreement with two statements “My sexual orientation is different from that of people around me” and “My sexual orientation is different from that of people close to me” (1 = *not at all*, 7 = *very much*) which were—as pre-registered—averaged into one score of sexual orientation deviance, *r*(465) = .89, 95% CI [.87, .91], *p* < .001.

Of the 467 pre-screened participants, 304 participants completed the experience sampling phase for in-situ reporting of ostracism experiences. For 14 days, participants indicated whenever they felt ostracized (i.e., an event-contingent measure of ostracism) using the app Expiwell (http://www.expiwell.com/). Moreover, individuals had the chance to report additional ostracism experiences every evening at 6 p.m. (i.e., time-contingently) to avoid underreporting.

### Results

#### Sexual Orientation

LGB individuals again descriptively reported higher retrospective ostracism frequency, *M* = 2.81, *SD* = 1.56, compared with straight individuals, *M* = 2.50, *SD* = 1.48, but the difference was not significant, *t*(173.76) = −1.86, *p =* .065, *d* = −.21, 95% CI [−.42, .01], η^2^ = .011. As depicted in Figure 1 (right graph), during the experience sampling phase, LGB individuals also descriptively reported more ostracism experiences, *M* = 3.82, *SD* = 4.87, than straight individuals, *M* = 2.82, *SD* = 3.39. The difference was not significant, *t*(93.44) = −1.63, *p =* .107, *d* = −.26, 95% CI [−.53, .00], η^2^ = .017.

#### Sexual Orientation Deviance

**Figure 2:**
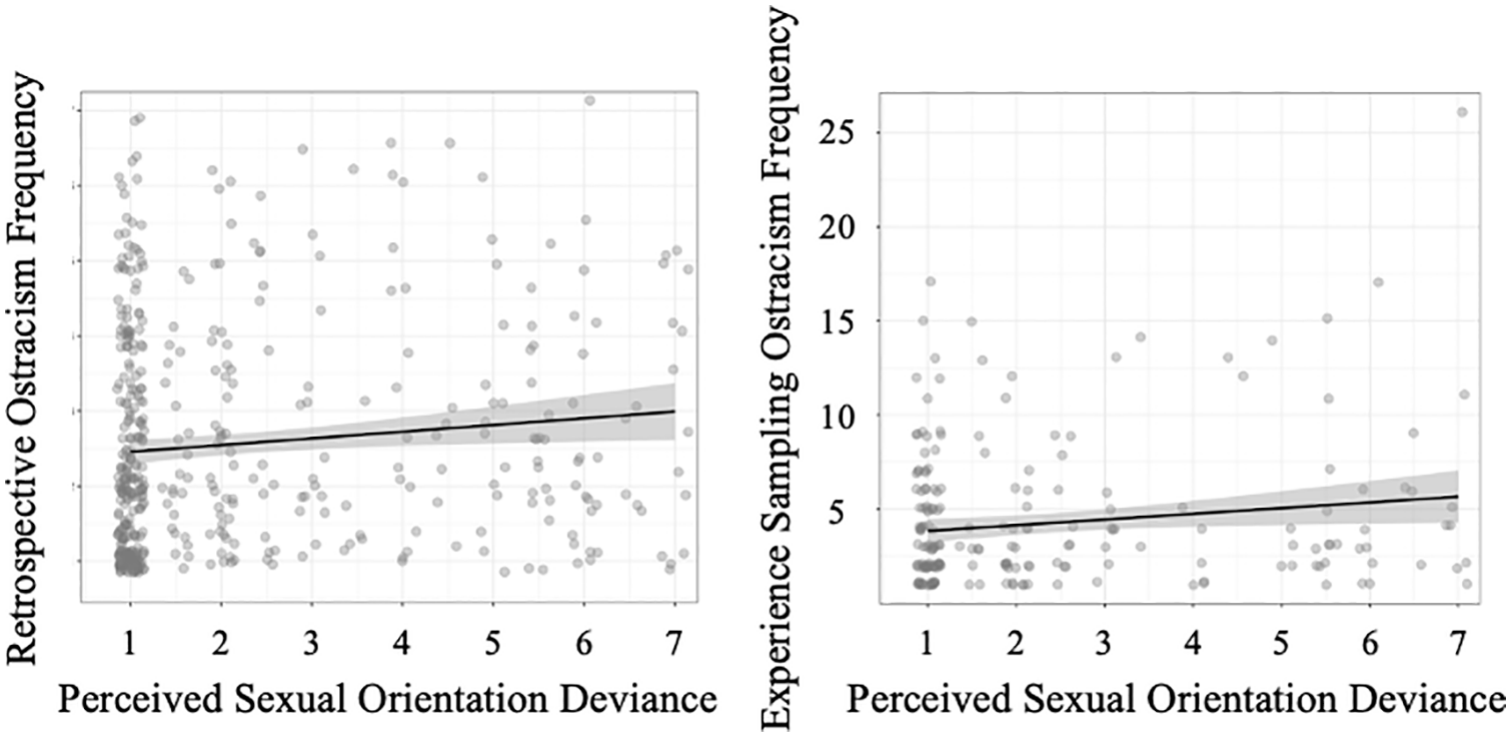
Sexual Orientation Deviance Predicting Retrospective Ostracism Frequency (Left Graph) and Experience Sampling Ostracism Frequency (Right Graph) in Study 2 *Note.* Gray areas around regression lines represent standard errors.

Sexual orientation deviance was significantly higher among lesbian/gay and bisexual individuals compared with straight individuals (see Table 2). Supporting H2, sexual orientation deviance was significantly associated with retrospective ostracism frequency, *b* = .09, 95% CI [.01, .16], *p* = .019, as well as with experience sampling ostracism frequency, *b* = .28, 95% CI [.05, .51], *p* = .018. Both associations are shown in Figure 2. Examined in exploratory fashion, sexual orientation deviance did not have significant effects on ostracism in the subgroups of lesbian/gay, bisexual, and straight individuals (see Table 2).

**Table 2.** Descriptive Statistics and Subgroup Analyses for Sexual Orientation Deviance in Study 2.

	Study phase			
	Pre-screening		Experience sampling	
	Sexual orientation deviance: *M, SD*, range	Subgroup analysis ostracism frequency by sexual orientation deviance	Sexual orientation deviance: *M, SD*, range	Subgroup analysis ostracism frequency by sexual orientation deviance
Lesbian/gay	5.29^a^, 1.21, 3-7	*b* = −.33, 95% CI [−.83, .17], *p* = .182	5.36^c^, 1.17, 3-7	*b* = −.68, 95% CI [−2.14, 0.78], *p* = .345
Bisexual	4.98^a^, 1.50, 1-7	*b* = .04, 95% CI [−.22, .31], *p* = .737	5.15^c^, 1.12, 2.50-7	*b* = .44, 95% CI [−0.85, 1.74], *p* = .492
Heterosexual	1.36^b^, 0.71, 1-7	*b* = .15, 95% CI [−.06, .37], *p* = .161	1.33^d^, 0.61, 1-4	*b* = .41, 95% CI [−0.31, 1.14], *p* = .263

*Note*. The letters a–d represent significant differences in means of sexual orientation deviance between groups obtained via Tukey’s post hoc tests. Means in the same column that share the same letter do not differ significantly from each other, means with different letters differ significantly.

### Discussion

Study 2 replicated results from Study 1 in showing that LGB individuals descriptively report more frequent ostracism experiences than straight individuals, both in retrospective recall and across an experience sampling period of 14 days. However, these differences were not significant. Sensitivity analyses conducted in G*Power 3 (Faul et al., 2007) revealed that sample size is unlikely to be the reason for the nonsignificant effects: Even though overall sample size was much larger in Study 1, LGB subgroups were small and thus the two studies are similar in their sensitivity to detect effects (i.e., for a *t*-test, Study 1: *d* = .28; Study 2: *d* = .27). The difference in the results of Studies 1 and 2 could be due to various reasons. First, context and time differ: While Study 2 was conducted in late 2022, Study 1 analyzes the social reality of LGB people in Germany over 7 years (2015–2022). Among other societal changes during this time frame, same-gender marriage was legalized in Germany in 2017 (Pew Research Center, 2023), an event that likely changed the social reality of many LGB people in Germany. Moreover, the samples considerably differ in sociodemographic characteristics: Participants in Study 2 were younger on average than participants in Study 1 (by 15-20 years, see Table 1). Younger individuals may experience ostracism more frequently than older individuals (e.g., Albath et al., 2023; Rudert, Janke, & Greifeneder, 2020) and suffer more from ostracism’s consequences (e.g., Pharo et al., 2011). Longitudinal studies also show age-related changes in the social reality of LGB individuals (e.g., Kneale & French, 2018). Therefore, investigating the reality of different age groups may have produced differences in the LGB *and* the straight groups between the studies. Furthermore, the studies investigate two different national contexts (Germany vs. the United States) that may be accompanied by different attitudes toward LGBs. For instance, in the World LGBT Equality Index, Germany takes 10th place in LGBT-friendliness, while the United States takes 25th place (World Equality Index, 2023).

Extending Study 1, Study 2 uncovered that self-reports of one’s own sexual orientation as different from those of others were strongly associated with retrospective reports and in-situ experiences of ostracism. This association could be due to many different reasons such as violating norms which is a powerful predictor of decisions to ostracize (Rudert et al., 2023). As previous research suggests, lesbians and gay men may be perceived as transgressing gender norms (e.g., Gordon & Meyer, 2007; Kachel et al., 2016) which leads to discrimination (e.g., Gordon & Meyer, 2007), and likely also ostracism. Thus, Study 3 tests explicitly whether conforming versus not conforming to gender roles drives ostracism of people varying in sexual orientation. Study 3 focuses on lesbian/gay and straight women and men only, without addressing bisexual people. This decision was based on previous research showing a strong heterosexual/non-heterosexual dichotomy: For instance, bisexual people self-stereotype as similarly gender-conforming as heterosexual people (Burke & LaFrance, 2016) and others perceive bisexual people as similar to same-gender lesbian/gay (instead of heterosexual) people (Rule & Alaei, 2016).

## Study 3

Going beyond Studies 1 and 2, Study 3 changes perspectives an investigates perceptions of ostracism, that is, how likely raters perceive that other people will ostracize a person based on that person’s sexual orientation and gender role nonconformity. Different stimuli (i.e., video with voice vs. video without voice vs. voice vs. full-body picture with head vs. full-body picture without head vs. head picture) from lesbian/gay and straight individuals (from here-on: targets) were rated by participants (from here-on: raters) regarding sexual orientation, perceptions of ostracism, and gender role (non)conformity.

We tested the following pre-registered hypotheses: Targets who self-identify as lesbian/gay will be rated as higher in perceived ostracism compared with targets who self-identify as straight (H3a). Based on previous evidence suggesting higher discrimination experiences for gay men compared with lesbians (Steffens & Wagner, 2004), we further test: Targets who self-identify as gay men will be rated as higher in perceived ostracism compared with targets who self-identify as lesbians (H3b). We also test the effect of other-rated sexual orientation: The more lesbian/gay a target is rated, the more that target is rated as likely to be ostracized (H4). Furthermore, we test the impact of self-ascribed and other-rated gender role (non)conformity on perceived ostracism: The higher the level of gender role nonconformity the targets ascribe to themselves (H5a), and the higher the level of gender role nonconformity the targets are ascribed by others (H5b), the more they will be rated as likely to be ostracized.^3^

### Method

#### Design

The study had three experimental factors: 2 (target gender: 36 female vs. 36 male) × 2 (targets’ sexual orientation: 36 lesbian/gay vs. 36 straight) × 6 (signal type: video with voice vs. video without voice vs. voice vs. full-body picture with head vs. full-body picture without head vs. head picture) with the first two factors varied within raters and the third factor varied between raters.

#### Targets and Stimuli

In total, 72 targets (18 lesbians, 18 gay men, 18 straight women, and 18 straight men) were rated; *M*_age_ = 24.17, *SD* = 2.26, Range = 20–30. For each target, six different stimuli were created in a controlled laboratory setting (see Kachel et al., under review, for more information). For dynamic stimuli (video with voice vs. without voice vs. voice only), targets were asked to give directions on campus while being video- and voice-recorded. For each target, we created three excerpts that were 3 to 5 seconds long. During each excerpt, targets looked into the camera at least once. None of the excerpts referred to sexual orientation, gender, or masculinity/femininity. For static stimuli (full-body pictures with head vs. without head vs. head only), targets were instructed to look directly into the camera with a neutral expression. Pictures were edited to be as comparable as possible: Targets were cut out and placed on a gray background. Color grading, brightness, and tone were standardized. Pictures without head were cropped and edited to include neither face nor hair.

#### Target-Assessed Variables

We assessed targets’ sexual orientation (lesbian/gay vs. straight), gender (female vs. male), and targets’ self-ascribed gender role nonconformity using the six-item Traditional Masculinity-Femininity scale (e.g., “I consider myself as . . .,” 1 = *very masculine*, 7 = *very feminine*, Kachel et al., 2016, Cronbach’s α = .91). To represent gender role nonconformity, scores for female targets were reverse-coded.

#### Raters

Raters were German speakers, at least 18 years old, from Clickworker (€4 for approximately 25 minutes). To reduce social desirability in raters’ responses, the study was advertised as a study of first impressions, without mentioning sexual orientation, and raters were unaware of targets’ self-identified sexual orientation. As pre-registered, we excluded one rater who did not provide informed consent, eight raters who displayed unreasonably fast reaction times, nine raters who reported having a visual disorder, and one who reported having a speech-, language-, or hearing disorder, as well as one rater who failed the attention check. In total, 141 raters who gave 10,760 ratings of perceived ostracism were retained in the sample: 68 women, 72 men, 1 diverse; *M*_age_ = 37.83, *SD* = 12.22, Range = 18–70.^4^

#### Rater-Assessed Variables

Our key dependent variable was ratings of perceived ostracism, one item: “This person is ostracized by others.” To avoid spillover effects, different groups of raters (applying the same exclusion criteria) provided ratings of targets’ sexual orientation (“This person is lesbian/gay”)^5^ and ratings of targets’ gender role nonconformity (“This person is gender conforming,” reverse-coded prior to analysis).^6^ All items were rated from 1 = *do not agree at all* to 7 = *do agree fully and completely*. In exploratory fashion, we assessed raters’ contact frequency with lesbians and gay men, one item each: “Frequency of contact with lesbians / gay men,” 1 = *never* to 7 = *constantly; M*_L_ = 2.81, *SD* = 1.40, *M*_G_ = 3.20, *SD* = 1.53, *r*(140) = .54, 95% CI [.41, .65], *p* < .001. We also assessed attitudes toward lesbian/gay individuals with the SABA scale (Preuß et al., 2020; higher values on a scale from 1 to 7 indicate more positive attitudes, *M* = 5.45, *SD* = 1.27).

### Results

#### Analytic Strategy

As pre-registered, following Barr et al. (2013), we computed multi-level models adding maximal random effects structure (i.e., random intercepts for raters and targets, random slope for predictor by rater, as well as random slope for signal type by rater). Because all models failed to converge, we removed (as pre-registered) the random slopes and report results from models with random intercepts only.^7^

#### Effects of Target Sexual Orientation and Gender on Ratings of Ostracism

**Figure 3.**
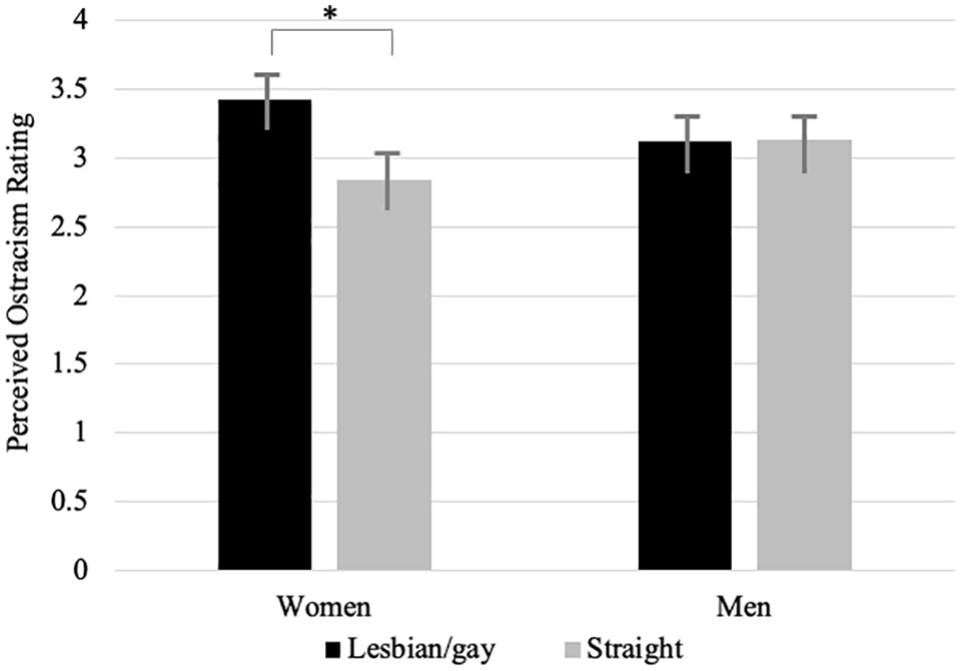
Sexual Orientation and Target Gender Predicting Perceived Ostracism Ratings in Study 3. *Note.* Vertical bars indicate standard errors of the means. *p < .05.

Descriptively, lesbian/gay targets were rated as higher in perceived ostracism, *k* = 4,940 ratings, *M* = 3.27, *SD* = 1.67, compared with straight individuals, *k* = 4,973 ratings, *M* = 2.99, *SD* = 1.56. Supporting H3a, a multi-level model indicated a significant difference, *b* = −.32, 95% CI [−.57, −.02], *p* = .037. To test whether gay men (vs. lesbians) are perceived as being ostracized more often (H3b), we probed for effects of target gender. Different from our predictions, lesbian targets were rated as higher in perceived ostracism, *k* = 2,470 ratings, *M* = 3.42, *SD* = 1.68, compared with gay targets, *k* = 2,611 ratings, *M* = 3.09, *SD* = 1.64. While the effect of sexual orientation (lesbian vs. gay) was not significant, *b* = .30, 95% CI [−.11, .70], *p* = .158, a model including target gender as an additional predictor showed a significant interaction of sexual orientation (lesbian/gay vs. straight) and target gender, *b* = .57, 95% CI [.04, 1.11], *p* = .040, as well as a significant effect of sexual orientation, *b* = −.58, 95% CI [−.96, −.21], *p* = .004, but no significant main effect of target gender, *b* = −.30, 95% CI [−.68, .08], *p* = .129. Tukey-adjusted simple effects testing showed that only the comparison of lesbians and straight women was significant, insofar that lesbians were rated as higher in perceived ostracism than straight women, *b* = .58, 95% CI [.08, 1.08], *p* = .014, all other *p*s > .388. Descriptive statistics are depicted in Figure 3.

#### Effects of Other-Rated Sexual Orientation on Ratings of Ostracism

Lesbian/gay targets were also rated as more lesbian/gay than straight targets, *t*(57.34) = 4.87, *p* < .001, *d* = 1.15, 95% CI [.64, 1.64] (*M_LG_* = 4.01, *SD* = 0.88; *M_straight_* = 3.17, *SD* = 0.53). Supporting H4, other-rated sexual orientation was significantly associated with perceived ostracism, *b* = .49, 95% CI [.37, .62], *p* < .001.

#### Effects of Gender Role (Non)Conformity on Ratings of Ostracism

**Figure 4.**
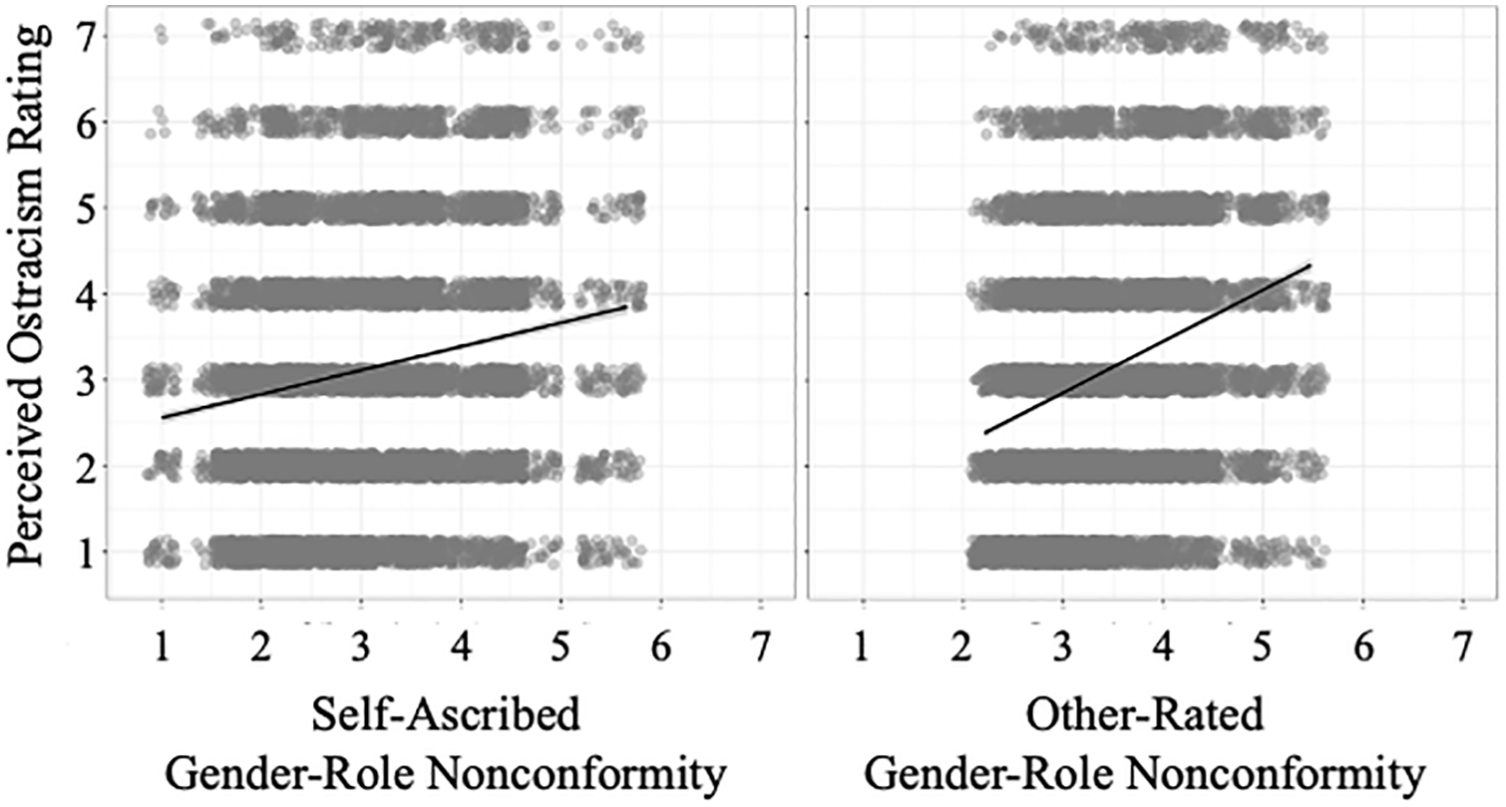
Self-Ascribed Gender Role Nonconformity (Left Graph) and Other-Rated Gender Role Nonconformity (Right Graph) Predicting Perceived Ostracism Ratings in Study 3. *Note.* Gray areas around regression lines represent standard errors.

Self-ascribed gender role nonconformity was significantly higher for lesbian/gay targets, *M* = 3.52, *SD* = 0.94, than for straight targets, *M* = 2.59, *SD* = 0.83, *t*(68.77) = 4.90, *p* < .001, *d* = 1.05, 95% CI [.55, 1.53]. Supporting H5a, targets’ self-ascribed gender role nonconformity was significantly associated with perceived ostracism ratings, *b* = .28, 95% CI [.15, .40], *p* < .001. Other-rated gender role nonconformity was significantly higher for lesbian/gay targets, *M* = 3.84, *SD* = 0.82, than for straight targets, *M* = 3.06, *SD* = 0.48, *t*(56.15) = 4.90, *p* < .001, *d* = 1.15, 95% CI [.65, 1.65]. Supporting H5b, other-rated gender role nonconformity was also significantly associated with perceived ostracism ratings, *b* = .60, 95% CI [.49, .72], *p* < .001. Self-ascribed and other-rated gender role nonconformity correlated significantly, *r*(70) = .56, 95% CI [.38, .70], *p* < .001. Both associations with perceived ostracism are shown in Figure 4.

#### Exploratory Pre-Registered Analyses

To identify the factors explaining most variance in perceived ostracism, we also ran a pre-registered model including target sexual orientation (straight vs. lesbian/gay), other-rated sexual orientation, self-ascribed gender role nonconformity, and other-rated gender role nonconformity predicting perceived ostracism ratings. Target sexual orientation, *b* = .26, 95% CI [.05, .47], *p* = .022, and other-rated gender role nonconformity, *b* = .68, 95% CI [.41, .94], *p* < .001, emerged as significant effects. Other-rated sexual orientation and self-ascribed gender role nonconformity were not significantly related to perceived ostracism in this model, *p*s = .350–.830.

We also pre-registered to probe for effects of target gender: For analyses of other-rated sexual orientation, self-ascribed gender role nonconformity, and other-rated gender role nonconformity, there were no effects of target gender, nor significant interaction effects between target gender and predictors, *p*s = .145–.991.

#### Exploratory Analyses (Not Pre-Registered)

To investigate the potential effect of psychosocial rater factors, we tested whether attitudes toward lesbian/gay individuals moderate the hypothesized effects. Indeed, attitudes toward lesbian/gay individuals interacted significantly with all tested predictors, specifically, the effect of all tested predictors was stronger for raters with more positive attitudes toward lesbian/gay individuals (except for the effect of identifying as lesbian versus gay: *p* = .107). See Table 3 for all inferential statistics.

**Table 3.** Exploratory Moderator Analyses of Attitudes Toward Lesbian/Gay Individuals, Study 3.

Tested predictor	Main effect predictor	Main effect attitudes	Interaction effect	Interpretation of interaction effect
Lesbian/gay vs. straight	*b* = .03, 95% CI [−.31, .37], *p* = .870	*b* = .04, 95% CI [−.08, .16], *p* = .526	***b* = −.06, 95% CI [−.10, −.02]**, *p* **= .002**	Perceived ostracism of lesbian/gay (but not of straight targets) is higher for raters with more positive attitudes.
Gay vs. lesbian	*b* = .05, 95% CI [−.45, .55], *p* = .850	*b* = .02, 95% CI [−.11, .14], *p* = .814	*b* = .05, 95% CI [−.01, .10], *p* = .107	n.s.
Other-rated sexual orientation	*b* = .17, 95% CI [−.01, .35], *p* = .059	***b* = −.21, 95% CI [−.35, −.06]**, *p* **= .006**	***b* = .06, 95% CI [.04, .08]**, *p* **< .001**	Effect of other-rated sexual orientation on perceived ostracism is stronger for raters with more positive attitudes.
Self-ascribed gender role nonconformity	*b* = .04, 95% CI [−.12, .21], *p* = .615	*b* = −.12, 95% CI [−.26, .01], *p* = .071	***b* = .04, 95% CI [.02, .06]**, *p* **< .001**	Effect of self-ascribed gender role nonconformity on perceived ostracism is stronger for raters with more positive attitudes.
Other-rated gender role nonconformity	***b* = .21, 95% CI [.03, .39]**, *p* **= .021**	***b* = −.24, 95% CI [−.39, −.09]**, *p* **= .001**	***b* = .07, 95% CI [.05, .10]**, *p* **< .001**	Effect of other-rated gender role nonconformity on perceived ostracism is stronger for raters with more positive attitudes.

*Note*. Significant effects are bold-faced.

We also tested whether more frequent contact with lesbian/gay individuals moderates the hypothesized effects. Contact frequency did not interact significantly with any of the tested predictors with one exception: The effect of sexual orientation (gay vs. lesbian) on perceived ostracism was stronger for raters with more frequent contact with lesbians and for raters with more frequent contact with gay men (see Table 4).

**Table 4.** Exploratory Moderator Analyses of Contact Frequency with Lesbian (L) and Gay (G) Individuals, Study 3.

Tested predictor	Main effect predictor	Main effect contact frequency	Interaction effect
Lesbian/gay vs. straight	*L: b* = −.29, 95% CI [−.58, .00], *p* = .050 *G: b* = −.28, 95% CI [−.57, .02], *p* = .067	***L: b* = .11, 95% CI [.00, .22]**, *p* **= .046** *G: b* = .03, 95% CI [−.06, .13], *p* = .482	*L: b* = −.001, 95% CI [−.04, .03], *p* = .961 *G: b* = −.01, 95% CI [−.04, .03], *p* = .695
Gay vs. lesbian	*L: b* = .13, 95% CI [−.30, .56], *p* = .553 *G: b* = .13, 95% CI [−.30, .56], *p* = .562	*L: b* = .08, 95% CI [−.03, .20], *p* = .164 *G: b* = .01, 95% CI [−.10, .11], *p* = .860	***L: b* = .06, 95% CI [.01, .11]**, *p* **= .020** ***G: b* = .05, 95% CI [.01, .10]**, *p* **= .024**
Other-rated sexual orientation	***L: b* = .48, 95% CI [.34, .61]**, *p* **< .001** *G*: ***b* = .45, 95% CI [.31, .59]**, *p* **< .001**	*L: b* = .09, 95% CI [−.04, .22], *p* = .191 *G: b* = −.02, 95% CI [−.13, .10], *p* = .781	*L: b* = .01, 95% CI [−.01, .03], *p* = .531 *G: b* = .01, 95% CI [−.01, .03], *p* = .163
Self-ascribed gender role nonconformity	***L: b* = .25, 95% CI [.11, .39]**, *p* ***<* .001** ***G: b* = .25, 95% CI [.11, .39]**, *p* **< .001**	*L: b* = .08, 95% CI [−.04, .20], *p* = .187 *G: b* = .005, 95% CI [−.10, .11], *p* = .931	*L: b* = .01, 95% CI [−.01, .03], *p* = .262 *G: b* = .01, 95% CI [−.01, .02], *p* = .275
Other-rated gender role nonconformity	***L: b* = .56, 95% CI [.43, .69]**, *p* ***<* .001** ***G: b* = .55, 95% CI [.41, .68]**, *p* ***<* .001**	*L: b* = .06, 95% CI [−.07, .19], *p* = .388 *G: b* = −.03, 95% CI [−.15, .09], *p* = .647	*L: b* = .02, 95% CI [−.01, .04], *p* = .180 *G: b* = .02, 95% CI [−.003, .04], *p* = .098

*Note*. Significant effects are bold-faced.

### Discussion

Study 3 offers novel insights into ostracism of sexual minorities: Lesbian and gay individuals not only report more ostracism (Studies 1 and 2) but are also perceived to face more ostracism by others. While targets’ sexual orientation (lesbian/gay vs. straight), and especially other-rated perceived sexual orientation, affected perceived ostracism ratings, the effects differed by gender: Lesbians were perceived to face more ostracism compared with straight women (no significant differences occurred when comparing the other groups). Previous research suggests that gay men face more homonegativity and discrimination compared to lesbians (e.g., Feinstein et al., 2012; Herek, 1988; Steffens & Wagner, 2004). Yet, in line with status beliefs theory (Ridgeway, 2001), it can be argued that lesbians encounter more discrimination due to being part of two marginalized, low-status groups (women *and* sexual minorities) compared with gay men who are only part of one marginalized group. Our findings on lesbians’ ostracism experiences align better with the latter theorizing; however, more research is needed to grasp underlying factors.

Although self-ascribed and other-rated sexual orientation, as well as gender role nonconformity were associated with perceived ostracism, other-rated gender role nonconformity ensued as the most important factor driving perceived ostracism. Correspondingly, our findings suggest that ostracism may not be confined to lesbians, gay men, and bisexuals but also straight people who are transgressing gender roles may be at risk of facing ostracism, while LGB individuals who conform to gender roles may not be, or, to a lesser extent. Ostracism may thus constitute a defensive threat reaction toward those who challenge gender roles and the gender binary (e.g., Glick et al., 2007; Morgenroth & Ryan, 2021; Van Der Toorn et al., 2020), irrespective of their self-identified sexual orientation. This aligns with findings that gender role nonconformity also detriments heterosexuals’ well-being (e.g., Tate et al., 2015).

Interestingly, the observed effects emerged for all signal types alike, suggesting that stigma information is transferred via multiple subtle cues. This aligns with the back-up-signal hypothesis that different cues such as faces and voices convey similar information (e.g., Smith et al., 2016).

Finally, some rater effects emerged: the influence of sexual orientation (lesbian/gay vs. straight, but not comparing lesbians and gay men), rated sexual orientation, and gender role nonconformity (self-ascribed and rated), on perceived ostracism were all stronger for raters with more positive attitudes toward lesbians and gay men. This aligns with previous findings that more positive attitudes toward minority members increase perceptions of minority discrimination among majority members (e.g., Bagci et al., 2017). The effect of sexual orientation (gay vs. lesbian) on perceived ostracism was stronger for raters with more frequent contact with lesbians and gay men. Just as intergroup contact increases support for social change among majority members (e.g., Hässler et al., 2020), potentially, more frequent contact with sexual minorities may sharpen individuals’ detection of discrimination, and, as a consequence, their perception of lesbians and gay men as more likely to face ostracism.

## General Discussion

Despite a myriad of studies on the discrimination of sexual minorities in general (e.g., Fasoli et al., 2017; Feinstein et al., 2012; Gordon & Meyer, 2007; Hebl et al., 2002; Katz-Wise & Hyde, 2012), research has been largely silent about the role of ostracism—an impactful form of discrimination that may strongly impact mental health and thriving of sexual minorities (e.g., Chen et al., 2020; Qian et al., 2019; Rudert et al., 2021). Compared with straight individuals, sexual minorities face more ostracism: This is shown in a nationally representative panel (*p* = .030, η^2^ = .001), also when participants were matched for age, gender, and income (*p* = .004, η^2^ = .07). Although nonsignificant results emerged from an experience sampling study of 14 days, descriptively, lesbian, gay, and bisexual participants reported more frequent ostracism experiences during the last two months (*p* = .065, *η*^2^ = .011), and during the experience sampling phase (*p* = .107, *η*^2^ = .017). As a first hint that deviation from heteronormativity may affect ostracism experiences, sexual orientation deviance significantly predicted higher ostracism frequencies. Applying an experimental approach by presenting stimuli of targets varying in sexual orientation, targets’ self-reported sexual orientation (lesbian/gay vs. straight), and perceived sexual orientation predicted perceived ostracism significantly. In addition, perceptions as likely targets of ostracism appeared to be gendered: Lesbians were rated as more likely to be ostracized by others than any other subgroup. Moreover, other-rated gender role nonconformity emerged as the strongest predictor of ostracism as rated by others, in line with the idea that people who challenge gender belief systems (Kite & Deaux, 1987) are ostracized as a consequence. Finally, in line with previous intergroup contact research (e.g., Bagci et al., 2017; Hässler et al., 2020), the effects were stronger (except for the comparison of lesbians and gay men) for raters with more positive attitudes toward sexual minorities, and the effect of sexual orientation (gay vs. lesbian) on perceived ostracism was stronger for raters with more frequent contact with lesbians and gay men.

So, what are the potential consequences of ostracism for lesbians, gay men, and bisexuals? Studies 1 and 2 showed small-to-medium differences in mean reported ostracism frequency between sexual minority members and heterosexuals (that were not significant in Study 2, see above). However, even small increases in ostracism frequency foster the development of depression and suicidality (Chen et al., 2020; Rudert et al., 2021). Consequences may be even stronger for LGB individuals because ostracism based on stable and internal attributes, such as sexual orientation, is particularly hurtful (e.g., Peng & Salter, 2021; Wirth & Williams, 2009). A common fallacy is that individuals are less impacted by adverse experiences if they are victimized over and over again (Zagefka, 2022), however, this is not the case for ostracism experiences, in fact, ostracism hurts the same over and over again (Büttner et al., 2023), and, even if individuals expect to be ostracized, ostracism still hurts (Jauch et al., 2022). Over time, ostracism may become chronic, causing individuals to enter the *resignation stage* (Williams, 2009), characterized by feeling hopeless, depressed, worthless (e.g., Riva et al., 2016), and unable to enjoy even positive interactions (Büttner et al., 2021, 2023). Certainly, this investigation is only a starting point to examine specific ostracism experiences that make up the social reality of LGB individuals and distinguish it from that of straight individuals. Future research should expand the present scope by analyzing LGBs’ ostracism experiences and, for instance, address (a) forms of ostracism specific to the LGB community (e.g., gender norm violations), (b) the impact of ostracism by straight individuals versus from within the queer community (e.g., in the case of bisexual erasure, see, for example, Morgenroth et al., 2022), and (c) factors hindering recovery from ostracism such as attributions to malicious sources’ motives (e.g., Goodwin et al., 2010). Protective factors against ostracism such as perceived social support and connectedness to the queer community should also be part of future research.

Another potential path explaining more frequent reports of ostracism that we did not address is that LGB individuals may *perceive* ostracism more readily. Drawing from workplace harassment literature (e.g., Nielsen & Knardahl, 2015), as well as the rejection sensitivity model of sexual minorities (Baams et al., 2020), frequent experiences of maltreatment may increase individuals’ readiness to perceive experiences as psychological maltreatment. Not all ostracism occurs out of the maliciousness of those who ostracize; people may also be overlooked, forgotten, or underrepresented unintentionally (e.g., Williams, 2009). Perceiving such instances as ostracizing and attributing them to intentional motives may increase the emotional consequences (e.g., Goodwin et al., 2010), as well as the perceived frequency of ostracism by LGB individuals. Longitudinal investigations should examine whether sexual minorities indeed develop a sensitivity toward ostracism (Baams et al., 2020).

Furthermore, insights accrued in the present research may inform interventions against queer discrimination by acknowledging ostracism as a form of maltreatment. Norms play an important role in decisions to ostracize others (see Rudert et al., 2023). Explicit acceptance of all sexual orientations in families, social circles, and organizations may set new norms that could aid in reducing ostracism of LGB individuals. There are also implications for allyship, that is, members of an advantaged group (i.e., straight people) who support matters of social justice for disadvantaged groups (e.g., Broido, 1997): When LGB individuals experience more frequent ostracism, allyship has to include being an ally against ostracism because failure to confront ostracism may aggravate the ostracized individual’s experience (e.g., Chernyak & Zayas, 2010; DeSouza et al., 2017).

This project paves the way for further queer ostracism research. For instance, future research should endeavor to shed more light on the experiences of transgender individuals who may be particularly at risk for ostracism (e.g., Peng & Salter, 2021) based on ostracizing experiences such as gendered language use (e.g., DeSouza et al., 2017; Stout & Dasgupta, 2011), being addressed with pronouns that they don’t identify with (e.g., Morgenroth et al., 2024), or transphobic microaggressions (e.g., Wesselmann et al., 2022). Future research could build upon the present findings to examine the degree of gender role nonconformity as a factor driving ostracism of transgender individuals. Future queer ostracism research should also include intersectionality effects, for instance, regarding age, race, or socioeconomic status.

Finally, we deem it important to note what our research does not imply. As a consequence of ostracism, LGB individuals may conceal their sexual orientation: In a study of LGB surgery residents, 57% concealed their sexual orientation because they feared ostracism (Lee et al., 2014). However, concealing one’s sexual orientation has harmful effects on well-being and life satisfaction (e.g., DeSouza et al., 2017; Herek, 2003; Quinn & Earnshaw, 2013). Therefore, while gender role nonconformity and perceptions of being different may shape LGB individuals’ experiences of ostracism, concealing one’s sexual orientation is not an implication of the present studies since it would wrongfully hold LGB individuals responsible and may ultimately cause more psychological harm.

## Conclusion

Ostracism is part of the social reality of LGB people and is mainly attributable to perceptions of gender role nonconformity. As gender role nonconformity is not confined to sexual minority members, ostracism may therefore also threaten straight individuals. We hope these findings pave the way for future research into ostracism experiences of queer individuals, allyship against ostracism, and intersectionality effects.

## Notes

1. This project focusses on lesbian/gay, and bisexual individuals.

2. Studies 2 and 3 were both part of larger projects. Here, we chose to focus on ostracism experiences and perceptions of ostracism of LGB individuals compared with straight individuals and only report measures relevant to the addressed questions.

3. We pre-registered to also test whether perceptions of ostracism by others are associated with higher frequency of ostracism experiences reported by targets. However, only lesbian and gay targets (*n* = 36) reported their ostracism experiences, therefore comparisons with straight targets are not possible. We report these analyses as supplementary in the R script (OSF).

4. We pre-registered to include raters’ sexual orientation (see OSF Supplement 1 for response options) as a potential control variable. However, as anticipated, subgroups were too small to run meaningful analyses (i.e., *n* = 11 of 141 raters indicated not being straight).

5. Other-rated sexual orientation rater sample: *n* = 87; 29 women, 58 men; *M*_age_ = 39.06, *SD* = 12.75, Range = 19-70.

6. Other-rated gender role nonconformity rater sample: *n* = 119; 46 women, 73 men; *M*_age_ = 40.64, *SD* = 12.35, Range = 18-67.

7. As pre-registered, signal type was a control variable in all analyses. Since signal type did not have significant effects in any of the reported analyses (smallest *p* = .440), see OSF for these effects.
